# *“We’re the very bottom, so it’s going to be hard for you to ‘catch any fish’ around here…”* understanding vulnerable Greenlanders’ perspectives on cancer and barriers to screening in Denmark– A qualitative study

**DOI:** 10.1186/s12939-024-02094-7

**Published:** 2024-01-22

**Authors:** Camilla Rahr Tatari, Berit Andersen, Pia Kirkegaard

**Affiliations:** 1 Department of Public Health Programmes, University Research Clinic for Cancer Screening, Randers Regional Hospital, Randers, Denmark; 2 Department of Clinical Medicine, Aarhus University, Aarhus, Denmark

**Keywords:** Mass screening, Early detection of cancer, Participation, Non-participation, Healthcare disparities, Vulnerable populations, Indigenous peoples, Qualitative research, Denmark, Greenland

## Abstract

**Background:**

Cancer is a major global health concern. Unfortunately, Indigenous populations such as Greenlanders living in Denmark, face significant disparities in cancer risk, incidence, diagnosis, care quality, and outcomes. In Denmark, vulnerable Greenlanders face challenges accessing cancer screening. The aim of this study was to explore their perceptions of cancer, barriers to participation in cancer screening, and potential for developing a tailored intervention.

**Methods:**

This qualitative study was based on participant observations and qualitative interviews. The sample comprised 46 participants from four distinct drop-in centres. Of these, 28 were vulnerable Greenlanders (19 women and 9 men), 9 were staff members (6 women and 3 men), and 6 were relatives (4 women and 2 men). The data were analysed through inductive content analysis.

**Results:**

Vulnerable Greenlanders in Denmark believed they were responsible for their own health and were generally satisfied with the healthcare system. However, they found it challenging to manage their own health and many depended on support from others. Fear of cancer and death shaped their attitudes towards screening.

**Conclusion:**

For vulnerable Greenlanders in Denmark participation in cancer screening programmes was positively viewed for most but could be challenging. Different intervention ideas raised by the vulnerable Greenlanders, relatives and staff members could guide the development of strategies to increase participation rates.

## Background

Cancer is a major global health concern and is responsible for a significant proportion of deaths worldwide [1]. To combat this, cancer screening programmes for breast, cervical and colorectal cancer have been developed in an effort to reduce both the incidence and mortality rates of cancer [2].

Indigenous populations are among the most vulnerable groups in the world. The United Nations Permanent Forum on Indigenous Issues has defined several characteristics for identifying Indigenous peoples, including self-identification, historical continuity with pre-colonial societies, a strong connection to traditional territories, distinct cultural and social systems, and a commitment to preserving ancestral environments and systems [3–5]. Many of these communities face significant health disparities, and cancer is one of the primary causes of illness and death for Indigenous populations. Efforts to address these disparities and improve Indigenous health outcomes are urgently needed [3, 6]. There are several barriers that Indigenous peoples may face when it comes to participating in cancer screening including cultural, linguistic, and geographic barriers. To address these barriers, a socially responsive approach to cancer screening is required. This approach involves working closely with Indigenous communities to gain an understanding of their needs and beliefs, and developing screening programmes that are tailored to meet those needs [4, 7–10].

Greenlanders are considered Indigenous people due to the history of Danish colonisation of Greenland. A smaller proportion of Greenlanders in Denmark face social vulnerabilities and social exclusion in the Danish society. Here vulnerability refers to adults who experience severe social problems, including homelessness, mental health problems, substance abuse issues, or/and loneliness. The challenges include language difficulties and a lack of knowledge about the Danish public system, including the healthcare system. Greenlanders in Denmark may not receive adequate attention to address these specific problems, leading to continued disparities in access to healthcare [11].

The aim of this study was to explore the perceptions of cancer and identify barriers to participation in cancer screening among vulnerable Greenlanders in Denmark. Additionally, we aimed to investigate the potential for developing a tailored intervention and relevant content to increase screening participation in this target group and reduce health disparities.

## Methods

### Design, setting and participants

The study design was based on qualitative research, incorporating participant observation and qualitative interviews.

All Greenlanders have Danish citizenship and therefore have free access to the three cancer screening programmes for cervical, breast and colorectal cancer and if needed free access to follow-up visits and treatment in Denmark [12]. All communication, including the invitation, the reminder invitation and other written information material about the screening programmes are in Danish. Participation is of course optional and can be actively opted out. Women are invited by digital mail to participate in cervical cancer screening every third year (aged 23–49) or every fifth year (aged 50–64). In the invitation the women are recommended to book an appointment at their general practitioner to have a cervical cytology specimen collected [13]. Most recently a self-sampling offer for Human papillomavirus (HPV) testing is provided if women did not participate after an invitation and one reminder. Biennial breast cancer screening is offered to women aged 50–69. The screening invitation comes with a pre-booked mammography appointment at a screening hub [14]. Finally, biennial colorectal cancer screening is offered both men and women aged 50–74. The screening consists of a faecal immunochemical test self-sample kit sent directly to the home [15, 16] with picture-based instruction on how to collect the sample and how to label it, and a preaddressed and prepaid return envelope [17].

About 17,000 Greenlanders live in Denmark and almost a fourth of them are either homeless (3.6%), on social security (11.2%) or on early retirement (8.8%) [18]. This study was conducted at four different drop-in centres located in two municipalities in Denmark. The drop-in centres cater to adults in a vulnerable position. The first centre is specifically for individuals over the age of 25, and approximately one out of five users are from Greenland. The second centre is exclusively for women, and approximately one out of three users are from Greenland. The last two drop-in centres are open to all but are targeted Greenlanders with or without severe problems, and approximately three in four users are from Greenland. The terms “user” (of the drop-in centre) or “Greenlander” will refer to “vulnerable Greenlander” throughout the paper.

The study’s inclusion criteria included both men (age 45–75) and women (23–75) of Greenlandic origin who were able to speak and understand Danish. To account for cognitive impairments and abuse-related issues, perspectives and experiences from staff members and relatives were included. This triangulation approach was chosen to ensure a more comprehensive understanding of the complex barriers being studied [19]. A total of 46 people participated in the study. Of these, 28 were Greenlanders (19 women and 9 men), 9 were staff members (9 women and 3 men), and 6 were relatives (4 women and 2 men). The term “participants” covers all who contributed with data to this study this includes Users/Greenlanders, relatives and staff members.

### Data collection

The Greenlanders were recruited for data collection through a snowball sampling strategy [20] at the different drop-in centres. The data collection was divided into two parts: Fall 2020 and Summer 2021.The collection process included participant observation, field notes, informal interviews, and semi-structured interviews. Prior to visiting each drop-in centre, the first author (CRT) talked with one or more staff members to present the study’s purpose and receive information about the centre, its users, and advice for interacting with them. CRT visited the centres mostly in the morning and afternoon, and one centre was visited from noon to night. During observations, CRT took breaks away from the users to record notes and observations. Each centre was visited two to three times for a minimum of eight hours each time. At the beginning and end of each visit, CRT held a small briefing with one or more staff members to hear updates since the last visit: any user comments, questions from the staff and the users, and overall feedback on CRT’s presence. These briefings were included in the field notes.

During the data collection, CRT received ongoing guidance and discussion from last author. Participant observation involved CRT participating in the day-to-day life at the drop-in centres, including monthly meetings for users and staff members, meals, and various activities. In addition, CRT also made informal interviews with users who were not comfortable being audio-recorded but wanted to contribute to the study in their own way. At the beginning of each conversation, CRT explained to the users why she was present and the purpose of the study. If they expressed interest in participating on their own terms, defined as giving verbal consent without audio recording, CRT proceeded to an informal interview, providing a briefing on their rights and the intended use of their statements. To maintain a comfortable environment and minimise distractions, only the notebook and relevant screening information materials were present on the table. During the informal interviews, the users allowed CRT to write notes and quotes. CRT memorised the interview guide (see Table 1) and selected some questions during the conversation to allow for flexibility based on the users’ mental state and to enable them to express what was important to them when talking about cancer and screening. The users were encouraged to freely express their perspectives and share their thoughts. The informal interviews were also conducted with relatives and staff members as the interviewee, depending on how busy the drop-in centres were and their feelings about being recorded.

Audio-recorded and semi-structured interviews were conducted with the participants. The participants were given the choice to select a location for the interview in a room with others or in a private room at the drop-in centre. Prior to the interviews, the participants were informed about the study’s purpose, and CRT emphasised that all perspectives were welcome and valued, regardless of whether they were positive or sceptical about cancer screening and participation in a tailored intervention. The participants verbally consented or signed a consent form and completed a questionnaire with background information. For the users, a semi-structured interview guide was developed by all authors and used (see Table 1). The interview questions were modified or omitted based on the user’s cognitive focus and the interview situation. Following both the informal and semi-structured interviews, a debriefing session was conducted to allow the users an additional opportunity to express their opinions. The interviews with relatives and staff members focused on three themes: (1) General experiences with vulnerable Greenlanders, (2) Health and prevention among vulnerable Greenlanders, including barriers, and (3) Perspectives on a tailored intervention, including its potential and content.

**Table 1:** Interview guide

Briefing	
PART 1: PERSPECTIVES ON CULTURE, OWN BODY, HEALTH, AND HEALTHCARE SYSTEM	
Topic	Question examples
Daily life	Can you tell me what a typical day looks like for you? How does life in Greenland differ from life in Denmark?
The Danish healthcare system	How do you usually feel about going to the doctor? How is it to talk to your doctor? Do you have access to digital mail?
Perceptions of health	What does being healthy mean to you? Who do you think is responsible for your health? (Why?)
Prevention	What does prevention mean to you? and how do you do it? Can someone be sick without feeling any symptoms?
PART 2: CANCER AND SCREENING	
Knowledge about Cancer	Why do people get cancer? What are the causes? Is it possible to prevent cancer?
Knowledge about Screening	Why do we screen? Do you think it’s relevant for you to participate?
*Optional: short introduction to cancer screening*	
Invitation and management	Can you tell me if you’ve received an invitation for a cancer screening? What do you think about using self-sampling kits for cancer screening? What do you think about the written materials that come with the screening invitation? (Here’s a brief explanation of the screening process and the materials you receive when you’re invited.)
Barriers	Can you describe what the screening process is like for you? Have you thought about getting screened? What worries or concerns do you have about getting screened? Do you feel like cancer screening is socially accepted in your community?
Tailored intervention	Would you be interested in participating? Where do you think changes could be made to improve participation? How can we communicate with you in the best way?
*Debriefing*	

In order to ensure an adequate sample size and information power, CRT shared and deliberated upon preliminary findings with staff members during the data collection [21]. Following this discussion, CRT and last authors evaluated the sample size before deciding to conclude data collection [21].

### Data analysis

All interview recordings and field notes were transcribed verbatim by CRT. A three-phase inductive content analysis was conducted to investigate the research aim based on the statements and experiences of the participants [22]. The analysis process consisted of preparation, organising, and reporting. During the first phase, CRT read the interviews carefully to gain an overall impression of the material. Field notes from the interviews and observations from the different drop-in centres were also used to contextualise the findings [22].

The second phase involved open coding, creating categories, and abstraction. This began with open coding of the transcriptions using NVivo 12, followed by grouping into subcategories, generic categories, and finally main categories. CRT made the open coding followed by grouping. The data grouping was presented to an extern researcher. CRT and the researcher discussed the process from open codes to the main categories [22]. Furthermore, this process was repeated with CRT and the other authors before completing the final main categories reported in the third phase [22].

### Ethical considerations

Given the vulnerability of the users in this study, we recognised our ethical obligations. Some Greenlanders faced cognitive challenges, and the staff recommended providing oral rather than written information about the study, as it is typically done. Additionally, the users were informed that they could withdraw from the interview at any time and were encouraged to take breaks as needed during the interview. At the end of each interview, the users were informed that they could contact the staff if they had any follow-up questions, wanted to contact CRT, or wished to withdraw their consent. If needed, the staff could then contact CRT on their behalf [23]. The ethical dilemma surrounding the potential harm to the Greenlanders was a central concern throughout the data collection process. The topic of cancer could evoke significant distress in some Greenlanders, conflicting with their avoidance strategy. In such instances, if strong emotions arose, CRT would offer options such as changing the subject, taking a break, or for the Greenlander to speak with a familiar staff member. Furthermore, CRT ‘s presence at the drop-in centres had been approved by the staff, specifically to address topics that could potentially distress some users [23, 24].

## Results

Five main categories that describe aspects of importance among Greenlanders to engage in cancer screening emerged from the data analysis: (1) Life as a daily struggle (2) Coping with the fear of cancer, (3) Embracing a mind-body-spirit approach, (4) From apathy to engagement, and (5) Unveiling the landscape of cancer screening needs and preference.

### Life as a daily struggle

The Greenlanders and the staff members explained how their life was characterised by instability and a sense of living from day to day. A staff member told how many felt unmotivated and struggled with procrastination as a coping strategy. Their physical form, mood, and energy levels could vary widely from day to day, sometimes from hour to hour or minute to minute. This variation made it difficult to plan and stick to a routine. Furthermore, they often experienced hunger and extreme fatigue. In terms of social status, they perceived themselves as being at the bottom of the hierarchy compared to other vulnerable groups, which aligned with the staff’s observations. Furthermore, they experienced loneliness, lack of strong networks and had a strong desire to belong to a community. Unfortunately, addiction often controlled their lives, making it challenging to break free from negative cycles. Moreover, they often lacked access to resources and support that could help them overcome their challenges. An example of this was illustrated in the following excerpt from the field notes. Here the project was just being introduced to an elderly user and he immediately commented on it: “He finds the project ‘commendable’, but he’s not interested in participating in it himself. He explains how he lives his life on the edge and therefore doesn’t see the point of participating. He’s going to die soon anyway– his own words. He says that he sees cancer screening as a ‘more beautiful form of self-harm’. He’s specifically referring to the waiting time for the result, which can make you sick in itself, a self-fulfilling prophecy. He describes himself and the others at the drop-in centre as the very bottom [of society], and he can’t see how we [the healthcare system] can ‘catch fish here’ unless we [the healthcare system] are very proactive and aggressive in our [the healthcare system’s] approach”. On the other hand, it was worth noting that the Greenlanders also displayed a concern about their health status, a positive attitude towards health and a keen interest in the screening programmes. A staff member explained it like this: “It’s not the first thing on their mind, it’s not. It’s not the second or third either. It’s significantly further down in their chaotic lives. But still, I think it’s definitely important for them. They should also be presented and informed about it, definitely”.

### Coping with the fear of cancer

#### Cancer is dangerous to talk about

For the Greenlanders in the study it was difficult to talk and discuss cancer openly. It was noted that they seldom talked about it amongst themselves. Those who had a family member or someone close to them affected by cancer found it easier to share their worries. An example on this could be seen in the excerpt from the field notes with a 40-year old woman: “She is worried about cancer because several women in her family have had it and died from it. She gets teary-eyed and expresses her deep concern”.

For most of the Greenlanders in the study, they tended to avoid thinking about it as they feared that such thoughts may have a negative impact on their lives. This perspective was expressed in this excerpt with a 72-years-old man: “He says he doesn’t think about cancer much, only when he hears about someone who has had it. He believes it can ruin your daily life”. Another man reacted with tears when he was introduced to the project, showing the fear of cancer and death in this excerpt: “I tell him about the project. He tells me straight away that he is not interested in participating in cancer screening. Silently he begins to cry, tears running down his cheeks. He says that he doesn’t want to know when he has to leave [die]. He tells me that he is too scared of a positive test result, that’s why he doesn’t want to participate. A few minutes after he falls asleep at the table”. The fear of being told they have cancer was a significant concern for the Greenlanders and was related to the fear of dying. Many Greenlanders hesitated to share their perspectives on the subject of cancer and screening due to this fear.

#### Cancer is a common disease

There was a general consensus among the Greenlanders that cancer was becoming increasingly prevalent. While alcohol consumption, smoking, and unhealthy eating habits were mentioned as possible causes, pollution and stress were believed to play a more significant role, as expressed in this quote by a woman in her fifties: “But I just think the world is so unhealthy, and the air is unhealthy. The earth is being polluted. Everyone’s exposed to it, and there are many who get cancer”. Another woman explained the development in cancer cases among Greenlanders like this: “I think it’s the food additives, like colorants and the lead content in food, especially in cans and such. Too many additives are not good. Greenlanders didn’t have as much cancer until they started consuming all those preserved canned foods. That’s when it started”. Moreover, it was emphasised that cancer was not a disease that could be prevented by a healthy lifestyle, as even healthy individuals could develop cancer. They believed that cancer could affect anyone but was more common with age, as a man explained it here: “Even if you have a good life with good eating habits, hardly any alcohol, don’t smoke,…they can still be affected by cancer. It’s been seen. So, even if you’re doing everything right, it [cancer] can still come out of nowhere”.

### Embracing a mind-body-spirit approach

#### Multifaceted health perspectives

Most Greenlanders expressed an interest in their health and many were concerned about their own health. Their focus on health included a focus on diet, social life, and stress avoidance. To maintain their mental well-being, they often avoided ‘bad thoughts’ like cancer, as exemplified by this woman: “Thinking positively is easier said than done. You need to be kind to yourself. Personally, I tend to be really tough on myself, and it varies based on how I’m feeling. I struggle with my illness and the abuse I faced as a child, and I try not to dwell on those things too much. As an adult, I’m responsible for creating a good life for myself, and fortunately, there are many choices I can make to stay healthy and happy”. The quote also showed how they perceived themselves as being responsible for their own health. Their thoughts about social life were illustrated in the quote with a middle-aged woman about prevention: “It’s about thinking healthy. Eating well and humanity, it means a lot. But we don’t talk about that much. [CRT: How does humanity matter?]. It’s about being important to others, and others feeling that you’re important. Yes, we are social animals, that’s how we’re wired”.

#### Choosing self-care over doctor visits

The Greenlanders explained how they were generally happy with the health system and it was one of the reasons for moving to Denmark. However, they often found it difficult to go to the doctor and preferred to handle their health themselves, as stated by a 44-year-old woman: “And if I have a headache, I could take painkillers, but I don’t bother. I mean, it’ll pass. We Greenlanders are very much like that, it’ll pass. We won’t die from it. But we have to learn to go to the doctor when we’re in pain. It’s even harder to go to the doctor when you’re not in pain”. They explained how it was common to deal with the pain themselves and they needed motivation from the staff members to see a doctor. A staff member explains it like this: “Some people have a good relationship and have had the same doctor for several years. And then there are those who don’t know who their doctor is. Those who are most challenged typically need extra motivation to go. Instead they try to ignore it [the pain] or explain it away”.

### From apathy to engagement

#### A shift in perspective

The Greenlanders had different attitudes towards cancer screening. Initially, most were uncertain about their view on the screening programme, thinking it was not for them. During the time they talked about the different topics they became more open to the idea and the relevance of cancer screening. Most ended up asking whether it was possible to participate right away. An example on this development was illustrated in the excerpt from a conversation with a man about screening: “He knows that he is invited to cancer screening. He is 60 years old and thinks that he has been invited a couple of times. He doesn’t immediately feel like participating, so he has just thrown it away. He says that the instructions are difficult to follow and it’s a tough test. He tells us that he managed to do it once. There he got help to position the paper [the piece of paper that is used to collect the stool] correctly on the toilet. He interrupts himself and asks if we can take the test now”. Others were interested from the beginning, like this woman stated it: “Screening is actually a pretty smart move. I know someone who didn’t participate, and cancer can be really aggressive - it can take you out pretty quickly”. Only a few could say with certainty that it was out of the question for them to participate.

#### Screening experience and the importance of support

Some Greenlanders had participated in colorectal cancer screening, like this woman recounting her experience with the stool sample: “There were these polyps. First there was this test where I had to send a sample to something or someone. Then they wrote that they had found blood in my stool. There is so much they can see in these things”. Although she further described the investigation and removal of polyps as painful, she intended to participate next time. On the other hand, some Greenlanders found the manual for completing the stool test too challenging due to the numerous steps involved, making the process ‘overwhelming and frustrating’, which discouraged some from completing the screening.

Some of the women told that they had previously participated in cervical cancer screening, mainly at the doctor’s suggestion, when they had to be examined for other reasons. Others mentioned how they became ‘curious’ when receiving the invitation and made appointments for themselves. A few women expressed discomfort with being examined and preferred to be examined by doctors with experience working with women like them. The women reacted positive towards the self-sampling for cervical cancer screening. However, most of them preferred to visit a doctor as they doubted their own ability to perform it correctly. A few also doubted the accuracy of the self-sampling test. Nevertheless, they overall expressed positivity about the test like one woman stated: “Wow, that’s nice!” and believed it would be beneficial for other women: “I would rather go to my own doctor. That’s what I would feel most comfortable with. But it’s a good idea. It could surely be good for many women”.

Some women have participated in breast cancer screening before and plan to do so again in the future. However, others found it too difficult to participate due to challenges such as transportation, scheduling, or a dislike of the screening method. For example, one woman expressed it like this: “Mammography? I don’t want to participate in that. [CRT: how come?] it’s because the hospital is so big - I would get lost, at least I think I would”.

Upon presentation of the screening information material, several Greenlanders expressed familiarity with its content and found the amount of information to be sufficient. Nevertheless, they preferred verbal communication and an option for written material in Greenlandic language. For many, understanding the benefits of early detection was enough. A few would like to learn more and discuss it with others. In regards to digital mail, most experienced how it was a challenge, as one middle-aged man expressed: “I can’t quite figure it out”. Some had even opted out of it entirely. While they appreciated the opportunity for cancer screening, they often encountered obstacles with the invitation and digital communication, particularly with digital mail, and relied on assistance to navigate these challenges. It was mentioned by several Greenlanders that they depended on staff members to support them in managing their health and/or to motivate them to take action. The staff members believed that if Greenlanders are motivated to participate, they will do their best to take the test, like one staff members said: " It’s more about whether they read it from the beginning and give up, but if they are motivated, I also believe they will complete it”.

### Unveiling the landscape of cancer screening needs and preference

The Greenlanders showed interest in participating in the cancer screening programmes supported by staff members and relatives. Table 2 presents the intervention ideas and preferences for a tailored screening intervention mentioned by the different participant groups.

**Table 2:** Intervention ideas and preferences presented by vulnerable Greenlanders, staff members, and relatives

Needs and preferences	Vulnerable Greenlanders	Staff members	Relatives
**Outreach initiatives**			
Face-to face engagement	X	x	x
Synergy with local experts		x	
Time allocation for trust establishment		x	
**Effective communication**			
Repetitive messaging	X		
Greenlandic language information material	X	x	x
Verbal communication	X		
Community-based group sessions	X	x	x
Heightened awareness	X		x
Enhance knowledge		x	
Q&A time	X	x	
**Other preferences**			
Safety-centric prioritisation		x	
Companion care inclusion		x	x
Self-sampling empowerment	X		

The staff raised a concern about the challenge of promoting preventive initiatives to a target group that lived from day to day and had limited resources, but they still believed in the importance of screening and other health initiatives for their users. The users and relatives shared this perspective and emphasised that these initiatives were also relevant for Greenlanders, even though introducing them might be difficult. As one 62-year-old woman put it: “If I’m interested, then I will participate. But right now, I don’t feel that way”.

#### Trust is essential

Although most Greenlanders were aware of the benefits of early cancer detection through screening, the subject was still difficult for them and often avoided. A staff member highlighted the importance of relationship-building and a comfortable environment, suggesting collaboration with a social nurse. Establishing trust and a safe environment should be the first step before implementing any intervention content. Therefore, any intervention should prioritise trust to encourage participation. According to the staff members this could be achieved through the involvement of familiar faces, such as people with experience working with Greenlanders, and local collaborations, which could help building trust.

#### Need for tailored communication

It was recommended to provide material in Greenlandic and repeat the same messages in a simple manner, keeping the information short and concise to make it more accessible to Greenlanders. A staff member suggested: “I think it would be helpful to have a brochure in Greenlandic that makes them feel that it is specifically for them and targeted towards them. This would capture their interest in a different way”. The Greenlanders were also keen for material in their own language, like a man expressed it: “I think that brochures could be a good idea and then just instruct the staff that there is a brochure about this, ‘just try to read about it’”. The staff would like to draw attention to the brochure as they have found that the various brochures placed at the drop-in centres were rarely looked at spontaneously. A woman summarised other preferences due to the communication strategy in this excerpt, stating: “She explains that the way to do it is through encouragement and information. It needs to be demonstrated and repeated many times: ‘Eventually it will sink in’”. Furthermore, she highlighted the importance of human interaction as a motivation strategy by saying: *“*Act like you [CRT] and me we do now. Away from the hospital, it should be humane”.

#### Group sessions and solidarity

Both Greenlanders and staff members mentioned that having a presentation on the subject at the drop-in centres would be relevant. By creating a safe space for open dialogue, it allowed them to engage in conversations about challenging topics among themselves. Many Greenlanders felt more comfortable participating in a group setting and believed that it can help reduce the stigma surrounding the topic. One man expressed his interest in a group session, stating: “If I am informed about it [a session], then I can see myself participate. If it’s an hour or two, the time is not a question, it’s just about making time and talking about it”. An extract from the field supported the interest in presentations: “As I’m talking with one user, another user approaches me who hasn’t been interested in talking with me before. She has an idea that there could be some lectures on the topic at the drop-in centre. She hopes that it will be possible in the future and that it won’t be too expensive”. In another interview, a woman expressed that group sessions could also have the advantage of bringing people together, saying: “I miss that we gather and talk about difficult things. It will be good for us. Cancer is dangerous, that’s for sure”.

#### The power of personal contact

The relatives highlighted the importance of an outreach strategy. In the following excerpt, a relative summarises what she thinks an intervention should be about: “She says that if I want to reach out to vulnerable Greenlanders, I need to meet them where they are. They need information, and preferably in Greenlandic. She explains that she participates in all the screening programmes she’s invited to and believes it’s important– even for the vulnerable. She doesn’t think there’s anything in the culture that would challenge their participation”. Both relatives, Greenlanders and the staff members emphasised the importance of face-to-face communication. Being present at the centres and showing interest was important. In an outreach approach it was also important to provide the possibility to participate right away. Most of the users who expressed interest in participating in cancer screening programmes were eager to do so immediately.

## Discussion

### Interpretation of results

This qualitative study represents, to the best of our knowledge, the first exploration of perceptions and experiences related to cancer and screening among Greenlanders in a highly vulnerable position. The Greenlanders included in our study confronted a multitude of challenges, including substance abuse issues that often resulted in cognitive difficulties, daily fluctuations in mental state, limited resources, and feelings of diminished self-worth and loneliness. Initially, the Greenlanders exhibited a tendency to avoid discussions and contemplation about cancer and their personal risk of being diagnosed with it. Their perspective stemmed from a viewpoint, wherein the mere thought of cancer had the potential to negatively impact their well-being. Some individuals had previously engaged in cancer screening, the majority demonstrated a familiarity of the benefits associated with early detection and many understood that screening offered an avenue for early identification of the disease. However, these individuals faced significant barriers to participation due to their life circumstances and social life. Nevertheless, a majority of the Greenlanders exhibited a desire to participate in screening. They expressed the need for regular awareness delivered through face-to-face interactions with compassionate individuals who genuinely cared about their welfare as a way of overcoming lack of motivation.

### Comparison to previous research

The decision to participate in cancer screening is often a complex and nuanced process that requires active decision-making. This process presents challenges for individuals with cognitive impairments and difficulties. This is similar to the experiences encountered by Greenlanders in this study due to alcohol abuse some are dealing with cognitive impairments and a living day to day approach to life. Such individuals commonly experience poor functional status, comorbid illnesses, and a shortened life expectancy [25–27]. A recent American survey study conducted in 2021 [27] aimed to explore the utilisation patterns of breast and colorectal cancer screening among individuals eligible for screening, taking into account their cognitive impairment status. The study found that individuals with cognitive impairment, whether mild or severe, were less likely to participate in cancer screening compared to those without cognitive impairment. These findings align with the barriers related to cognitive impairment identified in this study, highlighting the crucial need for social support in the decision-making process. Providing appropriate social support can play a vital role in facilitating their involvement in the decision-making process, ultimately improving their access to cancer screening [27].

The Greenlanders experienced fear of cancer, taboo about talking about the disease and there growing concern about the increasing prevalence of cancer among Greenlanders. A national and regional register study from 2016 [28] determined and compared the incidence of cancer among eight Arctic States with a special focus on three cross-national Indigenous groups, including Greenlanders. The study found an overall increase of cancer. Female breast cancer and colorectal cancer are increased significantly. Inuit men and women now face an overall cancer risk similar to that of non-Inuit populations in the United States, Canada, and Denmark [28]. A register study from 2018 [29] found that more Greenlanders are getting cancer, and their risk of dying from cancer is twice as high for Greenlanders compared to Danes and the other Nordic countries [29]. Furthermore, to nuance the perspective Hansen et al. found in a historical review of cancer diseases in Greenland that cancer was perceived as a “disease of civilisation” that did not exist in Greenland until the 1970s. It took a long time before it was recognised that even Greenlanders could get cancer [30]. This is in line with the perspectives of other Indigenous peoples concerning cancer and cancer screening as many Indigenous populations consider cancer as a new disease which they do not have a word for in their mother tongue [10].

The Greenlanders in this study demonstrated a keen interest in participation, particularly when they felt motivated, suggesting the potential efficacy of HPV self-sampling tests. A scoping review conducted in 2020 [31], focusing on HPV self-sampling in Indigenous communities, revealed that HPV self-sampling was perceived as easy, convenient, comfortable, and private. While the majority of experiences were positive, several studies indicated that some women lacked confidence in their ability to perform self-sampling adequately, aligning with the findings of this study. The review underscores the importance of incorporating supporting resources and community input to tailor the implementation of HPV self-sampling interventions to each community. This approach is crucial in maximising the effectiveness of HPV self-sampling in communities with low cancer screening participation, ensuring that the intervention is well-tailored and culturally appropriate for the specific needs and preferences of the community [31].

Many Greenlanders are dealing with lack of trust issues due to their experienced social position, limited network and unstable everyday life. In a realist synthesis conducted in 2020, specifically involving Indigenous patients [32], the aim was to gain a deeper understanding of how trust and world view function differently for Indigenous patients. The synthesis emphasised that the history and the impact of colonisation affected on the trust levels of Indigenous peoples towards institutions, as well as their distinct world view compared to non-Indigenous individuals. The study revealed that distrust and anxiety played a significant role and influenced the degree of engagement in a shared decision-making process negatively. This finding aligns with the results of this study, which also identified lack of trust as a barrier and showed the importance of establishing trust in any further work for this target group. Additionally, the synthesis emphasised the necessity for culturally appropriate support that is patient-focused and utilises the patient’s mother tongue. Implementing such support not only serves as a trust-building mechanism but also aids the decision-making process [32]. This further supports the findings of this study, which identified language barriers and a preference for material in Greenlandic.

### Methodological considerations

The authenticity of the quality of the data and the data collection were secured throughout the process of recruiting the users and the approach to the field. A major strength of this study lies in its data collection strategy and the participatory approach conducted at the drop-in centres, which provided a trusted and familiar setting for the Greenlanders. The data collection process successfully obtained complex and hard-to-reach data material. Despite being time-consuming, this approach effectively achieved its purpose by being confidence building and allowing Greenlanders the necessary time to express their perceptions and experiences regarding cancer and the screening programmes. It is worth noting that for most users, their cognitive difficulties posed challenges in reflecting on the subject. To address this, staff members and relatives were included in the data collection process, and collaboration with the staff members facilitated a better understanding of their first-hand experiences with the Greenlanders, including dos and don’ts for trustfulness interactions. Furthermore, the data was collected from multiple drop-in centres, enhancing the generalisability of our results. This approach secured data triangulation with multiple data sources to produce a more comprehensive view of cancer and screening and the inclusion of both perspectives from the Greenlanders, the staff and relatives. However, it is important to acknowledge that the findings may still be influenced by the specific context, setting, and the extent to which they align with the target group described in this study, particularly in terms of experienced barriers and facilitators. However, we believe that our results are likely to be transferable to other drop-in centres catering to Greenlanders or other Indigenous populations in vulnerable positions, experiencing severe social problems, within similar contexts [33].

The trustworthiness of the analysis was secured with the analysis process with a clear description of the roles of the research team and a systematic approach coding the complex data material. Furthermore an extern researcher contributed in the analysis served a neutral third party nuancing the results with her perspectives and understanding of the data material [33].

### Implications for practice

Based on the voices from vulnerable Greenlanders, their relatives and staff member there is an interest in participating in cancer screening. The key strategies and relevant content in an intervention should focus on the following strategies: trust building, awareness and outreach and access to screening. Establishing trust is vital. It can be done by collaborating with local key persons who are engaged in the community. This approach will bridge the gap between the screening initiative and the population. It is important to notice that cultivating trust demands patience and ongoing commitment. The information material should take into account their cultural, linguistic, and social needs. Furthermore, the material should resonate with their beliefs, addressing specific concerns. Face-to-face interaction should be prioritised to present the relevance of the cancer screening programmes. Finally, the access to screening could be improved by self-sampling kits or accompany arrangements to screening facilities.

## Conclusion

This study highlights vulnerable Greenlanders concern about their own health and interest in participation in cancer screening. The study indicates the need to address cancer disparities among vulnerable Greenlanders in Denmark. By understanding and addressing the challenges faced by this population, tailored interventions can be designed to reduce barriers and increase participation in cancer screening, ultimately leading to improved health outcomes for vulnerable Greenlanders.

## Data Availability

The datasets used and/or analysed during the current study are available from the corresponding author on reasonable request.

## Abbreviations

CRT: Camilla Rahr Tatari, first author
HPV: Human papillomavirus
